# Poisonous Plants and Flowers

**Published:** 1884-07

**Authors:** 


### POISONOUS PLANTS AND FLOWERS.

BUTTERCUPS possess a poisonous property which disappears when the flowers are dried. So caustic are the petals that they will sometimes inflame the skin of tender fingers. Every child should be cautioned against eating them; indeed, is is desirable to caution children about tasting the petals of any flowers, or putting leaves into the mouth, except those known to be harmless.

The oleander contains a deadly poison in its leaves and flowers, and is said to be a dangerous plant for the parlor or dining-room. The flower and berries of the wild bryony possess a powerful purgative, and the red berries, which attract children, have proved fatal.

The seeds of the laburnam and catalpa tree should be kept from children, and there is a poisonous property in their bark.

The seeds of the yellow and of the rough-podded vetches will produce nausea and severe headache.

Fool's parsley has tuberous roots, which have been mistaken for turnips, and produced a fatal effect an hour after they were eaten.

Meadow hemlock is said to be the hemlock which Socrates drank. It kills by its intense action on the nerves, producing complete insensibility and palsy of the arms and legs, and is a most dangerous drug except in skilful hands. In August it is found everywhere, and ladies and children gather its large clusters of tiny white flowers in quantities, without the least idea of their poisonous qualities.

The water hemlock, or cow bane, resembles parsnips, and has been eaten for them with deadly effect.

The water drop-wort resembles celery when not in flower, and its roots are also similar to that of the parsnip, but they contain a virulent poison, producing convulsions which end in death in a short time.

The fine-leaved water drop-wort and the common drop-wort are also dangerous weeds.

The bulbs of the daffodils were once mistaken for leeks and boiled in soup, causing sickness that lasted for several days.

